# Tobacco in post-conflict settings: the case of Iraq

**DOI:** 10.3332/ecancer.2017.735

**Published:** 2017-04-28

**Authors:** Zainab Hussain, Richard Sullivan

**Affiliations:** 1 King’s Centre for Global Health, Conflict and Health Research Group, King’s College London, London SE5 9RJ, UK; 2 King’s College London, Conflict and Health Research Group, King’s College London, London SE1 9RT, UK

**Keywords:** tobacco, conflict, policy, health, war, cigarette, smoking

## Abstract

Tobacco is the leading cause of preventable death in the world, claiming six million lives each year. Although smoking prevalence is decreasing in high-income countries, many low- and middle-income countries, particularly fragile and post-conflict nations such as Iraq, are still seeing prevalence rates rising. With the evidence of higher rates of nicotine dependence in conflict and post-conflict areas, the tobacco problem only becomes more difficult for Iraq, which finds itself involved in conflicts lasting years, if not decades. Terrorism and unstable governments, as well as Big Tobacco, create large roadblocks on the route to adequate tobacco control. However, some tobacco control efforts have been successful in other post-conflict settings, demonstrating that with good governance, many of these roadblocks can be overcome. This review explores the context of tobacco control strategies in Iraq, identifies problems/issues, and discusses possible resolutions using some examples from other post-conflict countries.

## Methods

**Figure 1.**
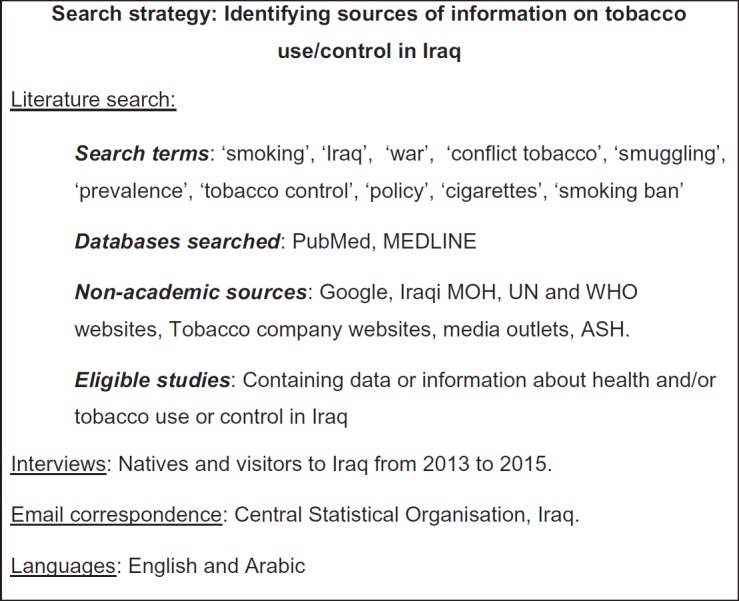
Methods.

The search strategy for this review is shown in Figure 1.

## Background

Approximately 1.1 billion people smoke worldwide, almost 80% of whom reside in low- and middle-income countries. With 26 countries now considered to be in active conflict or in the post-conflict setting [1], some 1.5 billion people are living in high exposure risk settings worldwide [2]. Evidence points towards links between conflict [3, 4, 5] and Post-traumatic Stress Disorder (PTSD) [6, 7] to tobacco use; as a result, these populations are much more likely to use, and/or be exposed to, tobacco. This can be attributed in part to the perceived stress relief of tobacco [3], and also to international tobacco companies using the weakened political situation in conflict zones to their advantage, by smuggling cigarettes [8] and shaping tobacco policy [9]. Increases in smoking prevalence are not limited to the civilian population; there is a greater than average prevalence of smoking in army recruits [10], which increases further in those who are deployed, and especially those with combat exposure [11].

In Iraq, a country that has seen several wars and conflicts over the last 30 years (Table 1), smoking prevalence estimates are estimated to be 31% and 4% for males and females, respectively [12]. The Global Youth Tobacco Survey (13- to 15-year olds) conducted in Kurdistan, Northern Iraq put smoking overall prevalence at 15.3%, 25.1% in men and 2.7% in women. In contrast, a similar Greek study where tobacco use is considered very high, estimated a prevalence rate of 10.4% [13]. Evidence shows that there is an increase in tobacco uptake during and post-conflict. This is attributed to increased stress levels and trauma, among other factors. However, other important causes are always present, including the breakdown of laws and government, and tobacco companies’ abuse of poor post-conflict governance in order to maximise profits [14].

Iraqis are mainly exposed to tobacco through cigarettes and shisha (water pipe tobacco). Pipes, cigars, and smokeless tobacco are not as popular. Furthermore, it is not culturally acceptable for women to smoke, which is reflected in the prevalence rates; more than six times as many men smoke than women [15]. Tobacco is both imported and exported from the country, with Iraq producing 4.4 billion cigarettes every year. Cigarette imports have increased since the start of the recent conflict, with a 50% increase between 2003 and 2005 [16].

Prior to the 2003 war, the country’s State Tobacco Company had the highest official share of the Iraqi market (although recent interviews with indigenous Iraqis suggest that this is no longer the case), with at least two factories in Baghdad alone. Their website advertises their product as being 100% Iraqi and ‘safer’ compared to imported cigarettes; ‘*most of the imported cigarettes contain high percentage from hydrizin material which cause cancer disease while the percentage in our cigarettes from this material is within the acceptable limit’* [17].

## Iraq and its failure to enforce the FCTC

The WHO Framework Convention on Tobacco Control has 188 signatories, out of the 193 United Nations member states, including Iraq [18], who signed the framework on 29 March 2004 [19]. In March 2012, Iraq put through new legislation on tobacco control, which included:

- ‘banning all forms of tobacco advertising, promotion and sponsorship
- requiring written and pictorial health warnings on all packaging of tobacco products
- banning the sale of tobacco products to and by minors;
- banning smoking in public places, including theatres, hotels, restaurants and shopping malls (but allowing designated smoking areas to be built within workplaces);
- providing incentives to tobacco growers to switch to alternative crops;
- *Raising public awareness of the impacts of tobacco on health, the environment and the economy, by introducing programmes targeting the general public through schools and health organizations’* [20]

Despite national legislation to enforce the FCTC, to date smoking cessation programmes and broader tobacco prevention measures have had little traction.

According to the 2011 WHO report on the Global Tobacco Epidemic [21], smoking cessation support is non-existent in Iraq; in hospitals and health clinics, or anywhere else in the community. Nicotine replacement therapy is available to buy from local pharmacies. Bupropion has been made available only recently, but varenicline is not legally available, either privately or on prescription [22]. This is not a problem limited to Iraq but is seen across the Eastern Mediterranean region. Thirteen per cent of the region has no cessation services, and 48% have minimal services or nicotine replacement therapy (not cost covered). Tobacco control programmes are not seen as a priority due to scarce recourses and lack of funding. The easiest way to implement smoking cessation support in a country such as Iraq, as recommended by WHO, is to integrate tobacco prevention into existing primary healthcare services. This can be seen as problematic, seeing as primary health care in Iraq is not up to standard. However, if smoking cessation strategies are integrated into existing systems, they will gradually improve as the health system is strengthened.

Iraq has varied scores for compliance to tobacco prevention strategies [22]. It rates highly on bans on tobacco advertising on TV, radio, and print, but there is low/no compliance with smoke-free environments, bans on billboard and outdoor advertising. Many of the prevention strategies listed within the document are not rated, presumably due to difficulty in acquiring such information. Health warnings are mandatory on cigarette packets, but there are no fines in place for violations (although compliance in this area is good; all packs sold in Iraq do have warnings on them). There are also no laws on misleading advertising, so tobacco companies can get away with advertising their product as ‘healthier’ than others (i.e., by using terms such as ‘low tar’, or as seen on the website for Iraq’s state tobacco company, claiming that their product contains less harmful carcinogens compared to imported cigarettes) [23].

Despite legislation in place that ban tobacco advertising and smoking in public places, billboards advertising cigarettes are up throughout the country [24]. In a 2008 study of students in Baghdad, 67.9% had seen pro-cigarette advertising on billboards, 67.6% had seen pro-cigarette advertising at point of sale locations, and 59.8% had seen pro-cigarette advertising in newspapers or magazines’ [25]. In 2015, WHO gave Iraq an average score of four out of 10, where 10 is top compliance, for control measures on direct tobacco advertising (television, magazines, billboards, etc.), and a score of four for indirect advertising (free distribution, promotional discounts, etc.) [22]. Although there are ‘no smoking’ signs up in hospitals and other public buildings, these are rarely enforced.

According to WHO and others, tax increases are ‘the single most effective intervention to reduce demand for tobacco’ [26]. Evidence from all resource settings has proven taxation to be an effective way to decrease smoking prevalence; price increases lead to greater numbers of people quitting tobacco and fewer people starting to use tobacco, although quitting is rarer in low- and middle-income countries [26]. Increasing the price of cigarettes by 10% leads to a 4% decrease in high-income countries. This percentage goes up to 8% in low- and middle-income countries [27].

**Figure 2.**
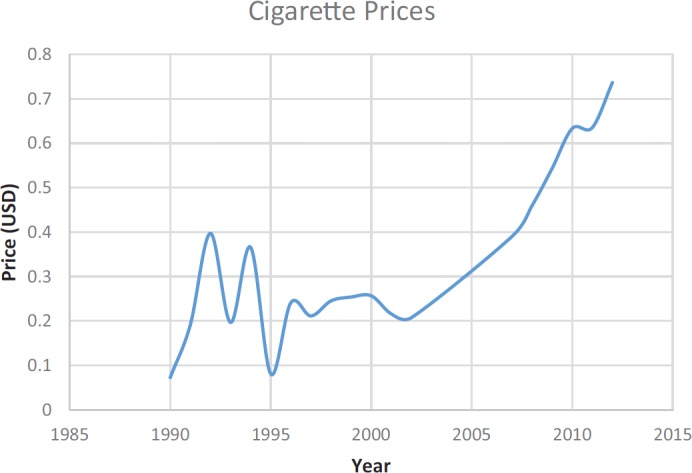
Changes in cigarette prices in Iraq from 1990 to 2012 [31].

At present, taxation on cigarettes in Iraq is minimal with only a small import duty; 19.24% as of 2014 [22]. No other taxes are levied on cigarettes, making the average cost of a pack of 20 just 0.78 USD in 2015 [28]. Prices have differed over the years (Figure 2), especially pre- and post-2003. However, cigarette prices in Iraq are among the lowest in the world; even with increases in taxation, a price increase of 140% from 2002 to 2012 only translates to a 0.53 USD increase in price. With Iraqi GDP at 5790.5 USD per capita [29], 78 cents for a pack of 20 cigarettes is extremely affordable, especially considering that there are cheaper brands available. The percentage of GDP required to buy 100 packs of the most popular brand is just 0.66% [22].

## Obstacles to successful tobacco control

Tobacco control interventions such as taxation, advertising bans *et al* do work, but the evidence is almost entirely drawn from high-income settings, with some rare examples in emerging economies [26]. As a post-conflict middle income country, the situation for Iraq is far more complex. The question is whether tobacco control is realistic in such settings given the multiplicity of political, security, social and economic factors stacked against such public health measures?

Although WHO does give Iraq average and high ratings for complying with some of its Framework Convention on Tobacco Control, the reality is that many tobacco prevention initiatives only exist on paper. Examples of this include the ban on smoking in public places and outdoor advertising; Iraq has policies in place for this but has failed to put them into practice. It is difficult to ignore the fact that the Iraqi state does gain revenue from sales and imports of cigarettes, via import duty and the SCTC (State Company for Tobacco and Cigarettes) and, with prevalence rates so high and so little competition, it can be assumed that the Iraqi government does not have much of an incentive to put real control mechanisms in place. However, if cigarettes were to be taxed appropriately, they would initially increase government revenue further, while also helping reduce the numbers of smokers. As government revenue is the major source of public health spending, a decrease in smokers now would arguably reduce the cost of healthcare spending in the future.

Another roadblock to overcome is tobacco smuggling, which is endemic to post-conflict environments [30]. Titeca *et al*. highlighted three main factors that help escalate the problem; the difficulty of monitoring borders in conflict settings, corrupt governments that allow smugglers to reign free, and cigarette smuggling being used to finance rebel groups. They draw attention to the fact that, unlike smuggling in countries such as the UK, smuggling in conflict settings is not influenced by differing taxation levels in surrounding countries, but the ‘outcome of weak state capacity, high levels of corruption and the activities of rebel groups’. Cigarette smuggling has been used in conflict the world over, from al-Qaeda and the PKK (Kurdistan Worker’s Party), to the IRA and CNDP (National Congress for the Defence of the People). With high profits (one container of 10 million cigarettes can bring in a profit of 1.9 million USD), and decreased risk due to its legal status, cigarettes are preferable to narcotics when it comes to smuggling. However, with the cooperation of governments and tobacco companies, it can be much easier to control smuggling of cigarettes compared to the smuggling of illegal substances by ensuring all cigarettes are sourced via legitimate channels. [31]

**Figure 3.**
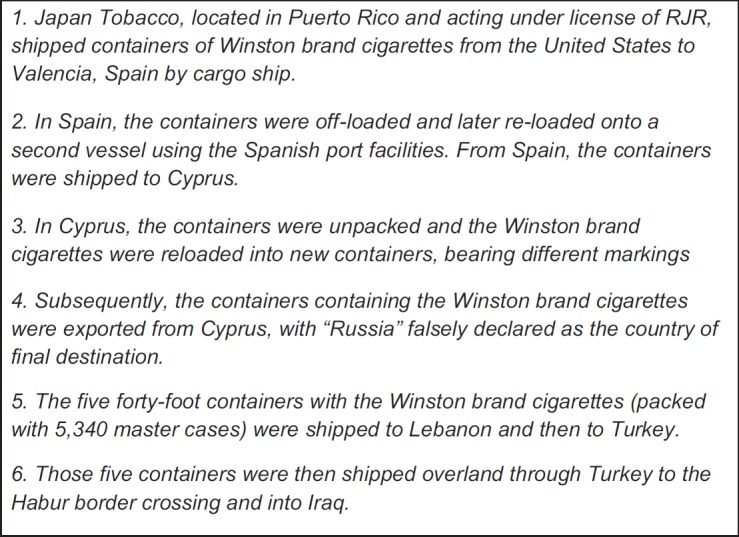
Steps of the scheme to smuggle cigarettes into Iraq (in place since at least August 1999) [35].

Illicit cigarette trade is a known problem in Iraq, but how deep does this issue go? In 2002, a law suit was filed by the European Union against two American tobacco companies, RJ Reynolds and Philip Morris, for their part in smuggling their tobacco products into Iraq, showing just how pervasive the issue had become with direct complicity by major tobacco companies [32, 33]. The evidence uncovered by the European Union demonstrated that the American tobacco companies had been illegally distributing their products to Iraq as early as the 1990s. Cigarettes would find their way to Iraq through complex transport routes; they would be packed in different containers and marks/numbers would be removed from products to prevent them from being traced (Figure 3). The report noted that, *‘Since 1996, approximately 50 billion cigarettes have been sent by RJ Reynolds Tobacco Company to Cyprus. Approximately half of these shipments were exported from Cyprus to Turkey in transit. Many of these shipments were destined for Iraq’* [32]. The law suit was dropped in 2004, after an agreement was reached between Phillip Morris and the European Union [34]. What is clear is that in post-conflict Iraq, with its low tax burden on cigarettes, what is driving smuggling is the competition between tobacco companies to increase market share [35].

## Beyond Iraq: post-conflict tobacco control across the world

Despite the obstacles that conflict brings, tobacco control in Iraq is by no means a lost cause. Other post-conflict countries have successfully implemented tobacco control policies.

### Iran

One such example is neighbouring Iran, which had been successful in implementing a complete ban on tobacco advertising in the 1990s, just after their eight-year war with Iraq [36]. What made this task slightly easier for Iran is that their tobacco industry was under state control and there was good collaboration across government and other sectors. The sale and import of tobacco products by foreign tobacco companies were not permitted, and so there was no real opposition to the ban by Big Tobacco. This, along with a health education programme and increases in tobacco taxes, resulted in a decline in smoking rates between 1991 and 1999 [37]. However, mostly due to transnational brands finding their way into the country via smuggling routes, Iran finally submitted to pressure and the tobacco industry was privatised. Predictably, these companies started opposing the advertising bans. Iran has seen some increase in prevalence rates since, although these have not been significant [38]. Iran still has good tobacco control measures in place and prevalence has remained fairly stable [38], with 20% of males and 2–3% of females smoking daily [39].

### Croatia

Four years after the end of the War of Independence, the Croatian Ministry of Health drafted a new law that was later passed through parliament as the Tobacco Product Use Restriction Act [40]. Prior to this, Croatia had weak legislation governing tobacco control, which included a ban on direct advertising of tobacco products. However, it failed to address indirect advertising, was poorly managed and fines for violations were small. Naturally, tobacco firms took advantage of this and continued their advertising campaigns.

The new act was vastly more detailed compared to previous legislation and addressed many of the loopholes that were previously exploited by tobacco companies and media. There was now a complete ban on all direct and indirect tobacco advertising in all except electronic media, which was not a significant issue at the time the law came to pass.

### Pakistan

Pakistan has a long history of conflict, starting with the first Indo–Pakistan war in 1947. Presently, the ongoing insurgency is causing thousands of fatalities a year. Between 2009 and 2010, with the technical assistance of WHO and Bloomberg Initiative funding, Pakistan increased cigarette taxation [41]. In 2013, Pakistan restructured their tobacco taxation, changing from a multi-tier to a more simplified two-tier system, where more expensive cigarettes have greater excise duty [42]. Although this will go some way to decrease tobacco use, the increased difference in price between the more expensive and cheaper brands will lead to many smokers switching to the cheaper brand of cigarettes, instead of decreasing cigarette sales overall. Pakistan, now on the way to better tobacco control, will need to continue in their efforts and make further changes to its taxation structure, shifting to the recommended single-tiered system, while adjusting tax rates yearly, with inflation.

### Vietnam

Vietnam has higher than average prevalence rates; about a quarter of the adult population smoke, mostly males [43]. After almost 20 years of conflict, the Vietnam War finally ended on 30 April 1975. Thirty years later, in 2005, Vietnam adopted the WHO Framework Convention on Tobacco Control (although their tobacco control efforts started in 1989 with the introduction of an advertising ban) [44].

Tobacco control efforts intensified in 2007, with the introduction of a new government, financial support of the Bloomberg Initiative and technical support of WHO, the Campaign for Tobacco Free Kids (TFK), and others. Prior to this, Vietnam had strong enforcement of advertising bans and health warnings on cigarette packets but performed poorly in enforcing their smoke-free laws. After 2007, the MoH worked closely with a myriad of organisation, managing to achieve high levels of compliance of the smoke-free regulations; schools, hospitals, and workplaces are virtually all smoke-free.

## Conclusions

There is no denying that tackling tobacco has been and will continue to be extremely challenging for Iraq in the post-conflict setting, made even more so by its weakened government and the involvement of international tobacco companies. Looking to other countries as examples: Iran with their successful advertising ban, and Vietnam’s smoke-free initiative, there is at least some evidence that tobacco control measures that have been successful previously can also be implemented in Iraq.

### Political will and leadership

Leadership, both in government, the Department of Health and in other organisations is essential in going forward with tobacco control legislation. Leaders will need to sustain enough political will to go through the processes that will take years and will be met with stiff opposition. [45] This opposition will come from the powerful tobacco companies, as well as from within the government and general population. Examples include arguments that tobacco control efforts deprive tobacco farmers and factory workers of their livelihoods, or arguments that tax increases lead to greater rates of tobacco smuggling. Regardless of the extent of truth to these claims, they will work hard to undermine tobacco control efforts, especially with the huge sums of money pumped into such advertising by Big Tobacco. Leaders across multiple agencies will have to work together to achieve a unified goal.

### Capacity building, technical support, and funding

As is the case in the majority of violent conflicts [46], the war in Iraq has resulted in large decreases in public revenue. Iraq has extensive public health and prevention programmes in place, and work is starting to be carried out to strengthen these. However, improvements are stunted by a rapid turnover of senior staff in the Ministry of Health, the ongoing instability and reliance on external donors for funding, among other issues [47]. As of 2014, there are no full-time staff working on the national tobacco control programme, though there is reportedly a small technical unit for tobacco control within the health system [48]. This presents obvious problems in strengthening the required infrastructure to proceed with legislation. Going ahead with legislation with limited capacity, with little to no means of assessing, implementing, monitoring, or evaluating outcomes, will be futile. For tobacco control legislation to be successful, necessary funding, human recourses, and technical support is required. For countries with limited capacity, this can be (at least partly) met by organisations external to the country. In the case of Vietnam and Pakistan, the Bloomberg Initiative provided both funding and technical support via their partners. Other organisations, including WHO, the Campaign for Tobacco Free Kids (TFK), The Union and the World Lung Foundation, Centres for Disease Control and Prevention (CDC) and the Gates Foundation can prove to be vital both financially and in terms of technical provision.

### Enforcing the law

Tobacco control efforts will never be effective without legislation being properly enforced by the relevant authorities. This too will require multi-agency cooperation; with enforcement by health authorities, law enforcement, and businesses. Enforcement will include monitoring, inspections, and reporting, with appropriate penalties for those who break the law. Croatia is a good example of this, successfully implementing stiff fines for those who break their advertising laws, forcing the tobacco industry to cease all direct and indirect advertising (except electronic media, which is an unfortunate loophole that needs to be addressed).

It will most likely take time, along with the significant effort and cooperation of all parties involved, from the ministries of health and education, to border control and security services, but Iraq may eventually be able to implement tobacco control measures that work as it one day exits the post-conflict era.

## Figures and Tables

**Table 1.** List of most recent conflicts involving Iraq.

Year	War
1980–1988	Iran–Iraq war
1990–1991	Gulf War
1991	Sha’aban Intifada
1995–1996	Iraqi Kurdish Civil War
1998	Operation Desert Fox
2003–2011	Iraq War
2014-date	Iraqi Civil War
